# Supplementary figures and images for: Prediction of hydrological and water quality data based on granular-ball rough set and k-nearest neighbor analysis
Source: PLoS One. 2024 Feb 23;19(2):e0298664. doi: 10.1371/journal.pone.0298664 (PMC10889668; doi:10.1371/journal.pone.0298664)

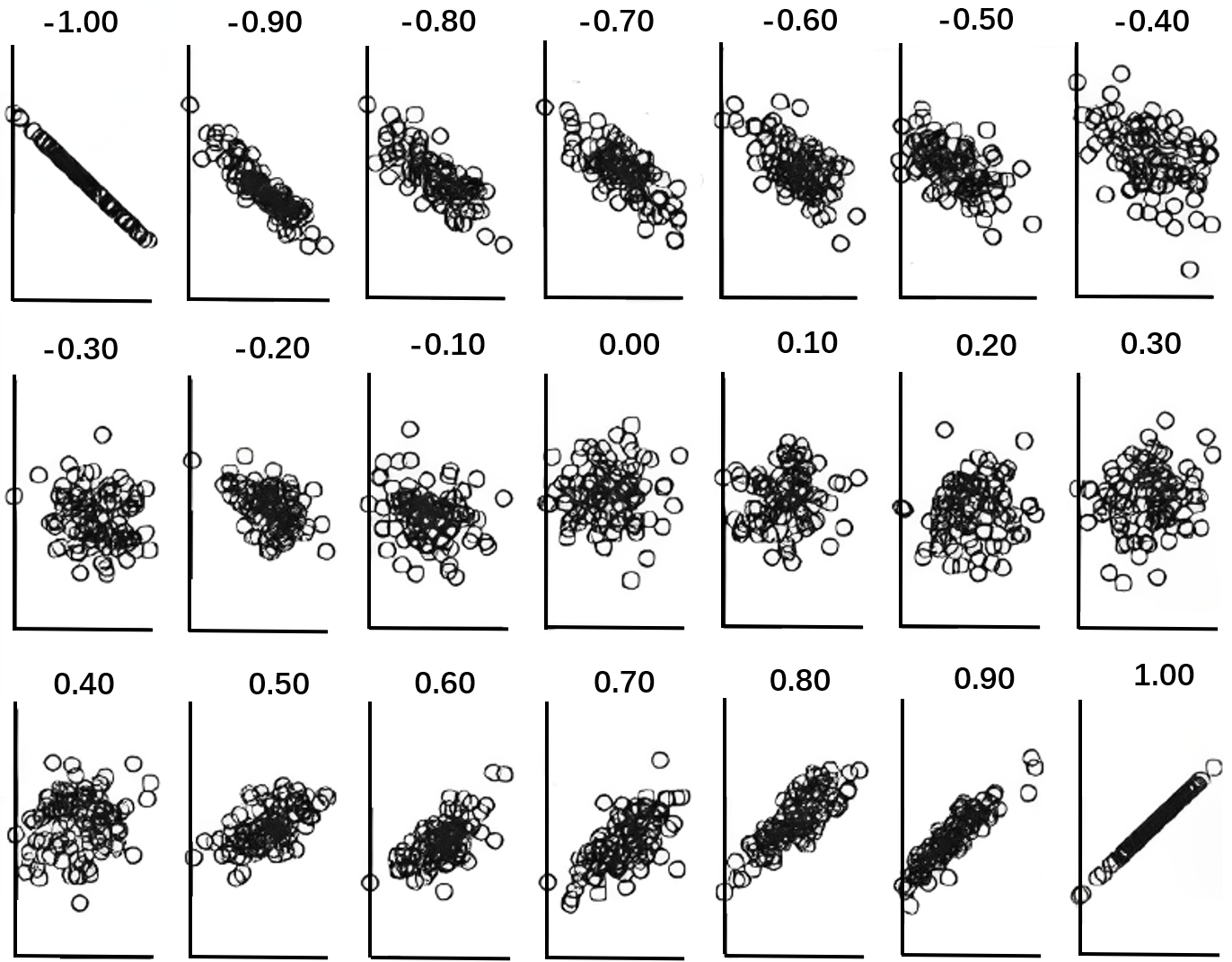

Supplement: S1 Fig — (TIF) [file pone.0298664.s001.tif]
