# Supplementary material for: Prediction of hydrological and water quality data based on granular-ball rough set and k-nearest neighbor analysis
Source: PLoS One. 2024 Feb 23;19(2):e0298664. doi: 10.1371/journal.pone.0298664 (PMC10889668; doi:10.1371/journal.pone.0298664)
Supplement: Attachment — Submitted filename: PONE-D-23-30013_Yasir_review.pdf [file pone.0298664.s002.pdf]

Prediction of Hydrological and Water Quality Data Based on Granular-ball Rough Set and kNN Analysis

General Comment:

The manuscript tries to combine the feature selection method as a role in hydrological and water quality analysis using the Granular-ball rough set and the kNN regressor, indicating that the feature selection method combined with the regressor can obtain better predictive learning results than a single regressor.

Specific Comments

1. The manuscript needs significant improvement in terms of language. A proofreading is required.
2. The research lacks novelty. The author needs to explain and emphasize more the innovation of this work in the introduction.
3. Starting of abstract seems not smooth, need major revision of abstract by including brief methodology, results, and findings of the manuscript.
4. Introduction section was written briefly, please add more literature about the research methodology. Furthermore, a comparison should be made over the operating conditions and yields in the literature rather than general information. The original aspect of the study should be emphasized. In addition, the aim of the study should be stated in more detail.
5. The methodology was not based on reliable references.
6. More explanation is needed for where there is a research gap and what the goals of the research are. The research gap and the goals of the research are not explained in detail, which leads to the reader missing the significance of the research.
7. The authors should compare the results with those of the previous researches, and this comparison should be presented in an individual table.
8. Extend the conclusions with all your most important findings.
9. The references need to be checked to make sure they are complete and up to date.

__Decision Letter__

I am pleased to inform you that the manuscript is accepted for publishing in your journal after editing for the minor revisions mentioned above.

Regards,

Dr.Yasir Al-Ani
