# Supplementary material for: Prediction of hydrological and water quality data based on granular-ball rough set and k-nearest neighbor analysis
Source: PLoS One. 2024 Feb 23;19(2):e0298664. doi: 10.1371/journal.pone.0298664 (PMC10889668; doi:10.1371/journal.pone.0298664)
Supplement: Attachment — Submitted filename: review of PONE-D-23-30013 manuscript.docx [file pone.0298664.s003.docx]

**Manuscript Number: PONE-D-23-30013**

**Title:** **Prediction of Hydrological and Water Quality Data Based on Granular-ball Rough Set and kNN Analysis**

**Comment 1**: Its better if the author follows the guideline of manuscript preparation of this journal (PLOSE ONE)

**Comment 2**: In abstract part, you didn’t state the objective of the research. It’s better if the statement of the problems, research gap and objective is stated very well. Also its better if you don’t use abbreviation in title and abstract part ‘**kNN**’

**Comment 3**: the study has no line number

**Comment 4:** in introduction part, first line its better if you cite after the end of the sentence.

**Comment 5:** the introduction part is very bulky. It needs to make it clear by modifying it. The research gap and objectives have to clearly provided in this part.

**Comment 6:** on page 3, the (equation…1) wasn’t cited. It needs to properly cite when you study scientific research.

**Comment 7:** in methodology part the research design have to properly stated. Therefore, it needs major revision and modification

**Comment 8:** on page 13, second paragraph discussion part, you didn’t discuss the limitation of your approach. It’s better if you clearly state the limitation on this study and also provide your recommendation.

Reviewer Name: Siraj Abduro Abdulahi

Email: [sirajab25@gmail.com](mailto:sirajab25@gmail.com)
