# Supplementary material for: Prediction of hydrological and water quality data based on granular-ball rough set and k-nearest neighbor analysis
Source: PLoS One. 2024 Feb 23;19(2):e0298664. doi: 10.1371/journal.pone.0298664 (PMC10889668; doi:10.1371/journal.pone.0298664)
Supplement: Attachment — Submitted filename: Response to Reviewers.docx [file pone.0298664.s004.docx]

Response Letter

✦

Dear Dharmendra Kumar Meena, Editors and Reviewers,

Enclosed please find the revised version of our paper “Prediction of Hydrological and Water Quality Data Based on Granular-ball Rough Set and k-Nearest Neighbor Analysis” with Manuscript ID: PONE-D-23-30013. We are very grateful to the editors and reviewers for your work on the review of this manuscript. The comments help us improve the quality of this manuscript. The paper has been revised with reference to Editors and reviewers’ comments and suggestions. We believe that the paper has been made more comprehensive and readable. Should you need further information, please let us know.

Yours sincerely,

Limei Dong, Xinyu Zuo, Yiping Xiong

## Response to Reviewer #1

*Title:* Prediction of Hydrological and Water Quality Data Based on Granular-ball Rough Set and k-Nearest Neighbor Analysis

*Manuscript ID:* PONE-D-23-30013

**Response to Each Comment**

**Note: Overall great literature with novel contributions.**

1-1) Granular-ball Rough Set not clearly explained in the introduction.

***Response:*** *Thank you very much for your great efforts on this manuscript, which will be of great help to us in writing higher quality articles in the future. Regarding the lack of clarity in the introduction of Granular-ball Rough Set, we have added relevant background information to the revised manuscript so that readers can better understand our research.*

***Action:***

***1.Introductin***

1-2) Repetition of “The basic idea of the kNN regression algorithm is to select the nearest training samples in K Euclidean spaces according to the similarity of Euclidean distance between samples, and the regression value is the mean or weighted value of K-nearest neighbor samples. ” under kNN regressor subsection.

***Response:*** *We thank you for pointing out the repeated statement of the kNN regression algorithm in the article. We have deleted the duplicated descriptions in the revised manuscript. Please see the revised draft for details.*

***Action:***

***2.3. kNN regressor***

1-3) BP neural network, what is BP? Could you make it more clear.

***Response:*** *Thank you very much for your great efforts on this manuscript.* *In the revised manuscript, we provide a more detailed explanation of BP neural network to ensure that readers can better understand the concept of BP neural network.*

***Action:***

***2.4.BP neural network***

## Response to Reviewer #2

*Title:* Prediction of Hydrological and Water Quality Data Based on Granular-ball Rough Set and k-Nearest Neighbor Analysis

*Manuscript ID:* PONE-D-23-30013

**Response to Each Comment**

**Comments:**

Comments to the Author The authors have addressed all the comments effectively.

**2-1)** The manuscript needs significant improvement in terms of language. A proofreading is required Could you make it more clear.

***Response:*** *Thank you very much for all your valuable suggestions to improve this manuscript. In the revised manuscript, we have conducted a comprehensive spell check and grammar proofreading to ensure that the language quality of the article has been significantly improved.*

**2-2)** The research lacks novelty. The author needs to explain and emphasize more the innovation of this work in the introduction.

***Response:*** *Thank you very much for all your valuable suggestions. We have strengthened the explanation and emphasis on the innovative nature of the work in the revised manuscript. The introduction of the article has been re-edited to more clearly describe the innovation and uniqueness of this study. Throughout the article, we include a separate section describing in more detail the new methods, new ideas, or new findings introduced by the study.*

***Action:***

***1.Introduction***

***3.Method***

**2-3)** Starting of abstract seems not smooth, need major revision of abstract by including
brief methodology, results, and findings of the manuscript.

***Response:*** *Thank you for your substantial contribution to this manuscript. In the revised manuscript, we have rewritten the abstract section to ensure a smoother start that quickly leads readers to the topic of the article. The abstract includes a concise overview of the research methods, a summary of key results, and a brief summary of the research findings to help readers better understand the content and contribution of the article.*

***Action:***

***Abstract***

**2-4)** Introduction section was written briefly, please add more literature about the research methodology. Furthermore, a comparison should be made over the operating conditions and yields in the literature rather than general information. The original aspect of the study should be emphasized. In addition, the aim of the study should be stated in more detail.

***Response:*** *Thank you very much for pointing out this problem. We have added and modified the descriptions of GBRS and BP neural networks in the introduction of the revised manuscript, and emphasized the contribution of this article. It will help to improve the level of detail and richness of the introduction to better guide readers into our research topic and emphasize the innovation and importance of the research.*

***Action:***

***1.Introduction***

**2-5)** The methodology was not based on reliable references.

***Response:*** *Thank you very much for your efforts on this manuscript, we describe the theoretical basis of our research methods in more detail in the methods section of the revised manuscript and cite relevant literature to support these theories. In addition, we improved the experimental design and analysis of experimental results to enable readers to better assess the credibility and reproducibility of our study.*

***Action:***

***1.Introduction***

**2-6)** More explanation is needed for where there is a research gap and what the goals of the research are. The research gap and the goals of the research are not explained in detail, which leads to the reader missing the significance of the research.

***Response:*** *Thank you very much for all your valuable suggestions. We describe the research background more clearly in the introduction section and particularly highlight the contributions of the work in this paper. Emphasize the significance of the research to ensure that readers can more clearly understand the contribution of our research to the field.*

***Action:***

***1.Introduction***

**2-7)** The authors should compare the results with those of the previous researches, and this comparison should be presented in an individual table.

***Response:*** *Thanks for your suggestions, we have recreated a dedicated table in the revised manuscript to summarize the key results comparison of PK and kNN methods. This table will provide an easy-to-read visual that allows readers to visually compare differences and similarities between studies. And the results of the comparison are explained in more depth in the results discussion section to help readers better understand the importance and contribution of the study. The detailed modifications are as follows.*

***Action:***

***4.3.Experimental Result Analysis***

**2-8)** Extend the conclusions with all your most important findings.

***Response:*** *Thank you very much for all your valuable suggestions. We have modified the table presentation in the revised manuscript to more clearly show the comparison of results between methods and ensure that they are highlighted in the conclusion section. Additionally, the practical and theoretical implications of our findings are discussed in detail in the results analysis to help readers better understand the importance of these findings to the field.*

***Action:***

***4.3.Experimental Result Analysis***

**2-9)** The references need to be checked to make sure they are complete and up to date.

***Response:*** *Thank you for your suggestion. Based on your suggestions, we checked and updated the references used, and we improved the introduction and supplemented the research progress of BP neural network and GBRS to make the article clearer and more complete.*

***Action:***

***1.Introduction***

## Response to Reviewer #3

*Title:* Prediction of Hydrological and Water Quality Data Based on Granular-ball Rough Set and k-Nearest Neighbor Analysis

*Manuscript ID:* PONE-D-23-30013

**Response to Each Comment**

**Note: The comments of the reviewer are in simple text, while our response is in *italic*. The modification we made in the revision is represented in the shaded boxes.**

3-1) Its better if the author follows the guideline of manuscript preparation of this journal (PLOSE ONE)

***Response:*** *Thank you for your suggestion. We have carefully studied the PLOS ONE journal's manuscript preparation guidelines and revised and adapted our article according to the requirements therein.*

3-2) In abstract part, you didn’t state the objective of the research. It’s better if the statement of the problems, research gap and objective is stated very well. Also its better if you don’t use abbreviation in title and abstract part ‘kNN’.

***Response:*** *Thank you very much for your great efforts on this manuscript. We have rewritten the abstract in the revised manuscript to clearly state the study's objectives, research questions, and research gaps. This will help readers better understand the motivation and purpose of our research. Additionally, we have avoided using the abbreviation "kNN" in the title and abstract, instead using the full name "k-nearest neighbor" to improve understandability and reduce confusion.*

***Action:***

***Abstract***

3-3) The study has no line number.

***Response:*** *Thank you for your suggestion.* *We 've added line numbers to the revision.*

3-4) In introduction part, first line its better if you cite after the end of the sentence.

***Response:*** *Based on your suggestion, we have added the corresponding reference to the first line of the introduction. The detailed modifications are as follows.*

***Action:***

1. ***Introduction***

3-5) Comment 5: the introduction part is very bulky. It needs to make it clear by modifying it. The research gap and objectives have to clearly provided in this part.

***Response:*** *Thank you very much for pointing out this problem. We have modified and improved the introduction, and emphasized the contribution of this article, research significance and chapter arrangement, etc., making the article clearer and smoother.*

***Action:***

1. ***Introduction***

3-6) Comment 6: on page 3, the (equation…1) wasn’t cited. It needs to properly cite when you study scientific research.

***Response:*** *We are very sorry that we did not cite correctly. In the revised manuscript, we have redescribed it and correctly cited the equations.*

***Action:***

***2.1. Granular-ball Rough Set***

3-7) Comment 7: in methodology part the research design have to properly stated. Therefore, it needs major revision and modification.

***Response:*** *Thank you very much for your enormous contribution to this manuscript. We have reorganized the work of this article into Section 3 and sorted out each method in detail to make the article clearer and smoother. Please see the revised draft for specific modifications.*

***Action:***

***3.Method***

3-8) Comment 8: on page 13, second paragraph discussion part, you didn’t discuss the limitation of your approach. It’s better if you clearly state the limitation on this study and also provide your recommendation.

***Response:*** *Thank you very much for your constructive suggestions. In fact, granular-ball computing is not suitable for high-dimensional and small sample data sets. We have added a description of this limitation in the conclusion section of the revised manuscript. The specific modifications are as follows.*

***Action:***

***6.Conclusions***

## Response to Reviewer #4

*Title:* Prediction of Hydrological and Water Quality Data Based on Granular-ball Rough Set and k-Nearest Neighbor Analysis

*Manuscript ID:* PONE-D-23-30013

**Comments to the Author**

 The manuscript, titled "Prediction of Hydrological and Water Quality Data Based on Granular Ball Rough Set and kNN Analysis," demonstrates technical robustness, offering a comprehensive overview of the employed methodology, and incorporates a meticulous statistical analysis. To enhance its quality, a more detailed introduction is recommended, focussing on comparing various prediction approaches or models, as the current content emphasises algal bloom in the introduction section.

**Response to Each Comment**

**Note: The comments of the reviewer are in simple text, while our response is in *italic*. The modification we made in the revision is represented in the shaded boxes.**

4-1) One notable gap in the paper is the lack of information regarding the method employed for data preprocessing. This crucial step in the research process should be elaborated on to provide transparency and enable replication by other researchers.

***Response:*** *Thank* *you very much for pointing out this problem.* *In the water quality data preprocessing method, we use the traditional analysis method of the Water Conservancy Bureau, such as the determination of water temperature using a thermometer or inverted thermometer method. The pH value was determined by the glass electrode method GB / T 6920-1986 and so on.*

4-2) Furthermore, the manuscript lacks a specification regarding the duration of the data periods studied. Clarifying the temporal scope of the data is essential for a thorough understanding of the research context and findings.

***Response:*** *Thank* *you very much for pointing out this problem.* *We have added the length of the study data period in the revised draft.*

***Action:***

***4.1.Sample Pretreatment***

4-3) Lastly, the absence of predicted time-series data for water quality is a notable limitation. Including this information would significantly enhance the completeness of the manuscript and contribute to a more holistic presentation of the study's outcomes.

***Response:*** *Thank* *you very much for pointing out this problem. We have supplemented the relevant instructions in the experiment.*

***Action:***

***4.1.Sample Pretreatment***

4-4) Addressing these gaps, namely, providing a detailed introduction comparing prediction approaches, elucidating the data preprocessing method, specifying the duration of data periods, and incorporating predicted time-series data for water quality, will significantly improve the manuscript's overall quality and render it suitable for publication.

***Response:*** *Thank* *you very much for pointing out this problem. We have supplemented the relevant instructions in the experiment.*

***Action:***

***4.1.Sample Pretreatment***

## Response to Reviewer #5

*Title:* Prediction of Hydrological and Water Quality Data Based on Granular-ball Rough Set and k-Nearest Neighbor Analysis

*Manuscript ID:* PONE-D-23-30013

**Comments to the Author**

 This paper proposes a methodology of synergizing Granular-ball rough set analysis with kNN regression to dissect hydrological water quality data. The paper is very well written, which enables to Improve the accuracy and reliability of hydrologic and water quality analysis.

**Response to Each Comment**

**Note: The comments of the reviewer are in simple text, while our response is in *italic*. The modification we made in the revision is represented in the shaded boxes.**

5-1) In INTRODUCTION, the latest relevant research background needs to be supplemented. The research background of the data analysis method and regression analysis algorithm used in this paper in hydrology and water quality is rarely introduced, and there is also a lack of new literature. How the study relates to this previously published research and how the model approach you used differs from previous research?

***Response:*** *Thank you very much for your contribution to this paper. We have supplemented the latest relevant research background in the introduction, and added the research background of regression analysis algorithms in hydrology and water quality as well as the research of GBRS, emphasizing how this research is related to previously published research, and the significance of this work. The main contribution makes the entire article clearer.*

***Action:***

***1.Introduction***

5-2) Figure 2, Figure 3, and Figure 4 all show a flow cahrt of the method usd in this paper, and authors should consider a more comprehensive and concise presentation.

***Response:*** *We are very sorry for the unclear description. In the revised manuscript, we have listed the method of this work as a separate section 3. Figure 2 is the process of BP neural network. Figure 3 describes the overall flow chart of the Pearson correlation coefficient combined with the regression method. Figure 4 describes the process of GBRS combined with regression method in detail. We have reworked the introduction of the methods in this article to make it clearer and more complete.*

***Action:***

***3.Method***

5-3) From Table 2 to Table 8, the authors use a total of eight tables to show the mroot mean square error, suggesting a more concise display method or a choice.

***Response:*** *Thank you for your suggestion. In view of the redundancy of the tables, we have compressed the tables, leaving the necessary tables to display the results, and re-analyzed the results of the relevant tables. The specific modifications are as follows.*

***Action:***

***4.3.Experimental Result Analysis***

5-4) Table 7 and Table 8 respectively introduce the root mean square error results using KNN regression model and BP neural network, but the content formats of table header are not uniform.

***Response:*** *Thank you very much for pointing this out. In our revised manuscript, we compressed the original redundant tables, and the original Table 8 was replaced by Table 4. Three evaluation indicators, MAE, MAPE and R^2, have been added to the BP neural network result table. Table 7 shows the comparison of the results of PK and kNN under different values of k. Furthermore, we reanalyzed the tabulated results accordingly.*

***Action:***

***4.3.Experimental Result Analysis***

5-5) Most of the table formats need to be adjusted, for example, the header lines in Tables 1 to 7 are too high.

***Response:*** *Thank* *you very much for pointing out this problem.* *We have deleted the redundant tables and modified the format of the tables. The specific modifications are as follows.*

***Action:***

***4.3.Experimental Result Analysis***

5-6) Is it limited to use only root mean square error as an evaluation index to predict model performance? Why not use more evaluation indexes for comprehensive analysis?

***Response:*** *Thank* *you very much for pointing out this problem. The evaluation indicators are not limited to the root mean square error. For the BP neural network method experiment, we added MAE, MAPE, and* $R^{2}$ *indicators to evaluate the results and conduct analysis and discussion. The detailed modifications are as follows.*

***Action:***

***4.2.Evaluating Indicator***

***4.3.Experimental Result Analysis***

5-7) This paper only mentions the sources of water level, flow, water quality and ecological data, which should be supplemented with specific information on hydrology and water quality.

***Response:*** *Thank* *you very much for pointing out this problem. We provide more detailed information, including specific sources of hydrological and water quality data, in the methods section. This will include details on where the data is collected, how often it is collected, and the agency from which the data is sourced.*

***Action:***

***4.1.Sample Pretreatment***

5-8) Two different algorithms, KNN regressor and BP neural network, and two data analysis methods, Granular-ball Rough Set analysis and Pearson correlation coefficient, used in this paper have been widely used in many fields. In this paper, different algorithms and data analysis methods are combined respectively to analyze, and the main conclusion is that the combination of Granular-ball Rough Set analysis and kNN regressor better results than using independent kNN regressor. First of all, the results of independent use of kNN regressors are not included in this paper. Secondly, simultaneous innovation has not been explained and highlighted in detail. Whether the conclusions are universal, and whether the data results are different in different places or time periods.

***Response:*** *We are very sorry for the unclear description. We have presented results on using the kNN regressor independently in the article to provide more comprehensive findings. This will include detailed results presentation and discussion so that readers can understand the performance and contribution of the kNN regressor in the study. In addition, we explain and highlight the innovative aspects in more detail in the revised manuscript to ensure that readers understand the innovations in the research methods and results. More evaluation indicators have also been added to illustrate the effectiveness of this method.*

***Action:***

***2.3. kNN regressor***

***4.3.Experimental Result Analysis***

5-9) The format of the references is inconsistent, such as the 45th reference and the 53rd to 59th reference.

***Response:*** *Thank you for pointing out this error. We have checked all references and corrected all errors.*

***Action:***

***References***

## Response to Reviewer #6

*Title:* Prediction of Hydrological and Water Quality Data Based on Granular-ball Rough Set and k-Nearest Neighbor Analysis

*Manuscript ID:* PONE-D-23-30013

**Comments to the Author**

 This paper proposes a methodology of synergizing Granular-ball rough set analysis with kNN regression to dissect hydrological water quality data. The paper is very well written, which enables to Improve the accuracy and reliability of hydrologic and water quality analysis.

**Response to Each Comment**

**Note: The comments of the reviewer are in simple text, while our response is in *italic*. The modification we made in the revision is represented in the shaded boxes.**

6-1) The abstract needs revision. It is always better to provide quantitative results in the abstract. The conclusion mentioned in abstract is too generic 'superior result' rather than in what term prroposed approach was superior to KNN would be meaningful.

***Response:*** *Thank* *you very much for pointing out this problem. In the revised manuscript, we will rewrite the abstract section to ensure a smoother start that quickly leads readers to the topic of the article. The abstract includes a concise overview of the research methods, a summary of key results, and a brief summary of the research findings to help readers better understand the content and contribution of the article.*

***Action:***

***Abstract***

6-2) Figure 1. I don't see the essence of Figure 1. It can be moved to annex or supplementary section.

***Response:*** *Thank* *you very much for pointing out this problem, we have moved Figure 1 to the attachment based on your suggestion.*

***Action:***

6-3) Page 7: In both the subsection you have mentioned about the proposed flow chart in this article. However, the title shows that your proposed methodology is granular ball and KNN regression. Please justify.

***Response:*** *Thank* *you very much for pointing out this problem.* *We are very sorry for the unclear description. In the revised manuscript, we have listed the method of this work as a separate section 3. Figure 2 is the process of BP neural network. Figure 3 describes the overall flow chart of the Pearson correlation coefficient combined with the regression method. Figure 4 describes the process of GBRS combined with regression method in detail. We have reworked the introduction of the methods in this article to make it clearer and more complete.*

***Action:***

***3.Method***

6-4) The paper has too many tables. Try to reduce to 4 numbers rather than 8.

***Response:*** *Thank* *you very much for pointing out this problem.* *In view of the redundancy of the tables, we have compressed the tables, leaving the necessary tables to display the results, and re-analyzed the results of the relevant tables.*

***Action:***

***4.3.Experimental Result Analysis***

6-5) I am not sure whether the authors are clear on the works they are doing. Somewhere they have mentioned about hydrology and water quality and somewhere hydrology water quality (in abstract). Be consistent on the work you are doing. What difference do the author want to portray from these discrepancies, if there are any.

***Response:*** *Thank* *you very much for pointing out this problem.* *We have supplemented the latest relevant research background in the introduction, and added the research background of regression analysis algorithms in hydrology and water quality as well as the research of GBRS, emphasizing how this research is related to previously published research, and the significance of this work. The main contribution makes the entire article clearer.*

***Action:***

***1.Introduction***
